# Research Progress of Sodium Alginate-Based Hydrogels in Biomedical Engineering

**DOI:** 10.3390/gels11090758

**Published:** 2025-09-20

**Authors:** Juan Cao, Bo Wu, Ping Yuan, Yeqi Liu, Cheng Hu

**Affiliations:** 1 School of Fashion and Design Art, Sichuan Normal University, Chengdu 610066, China; j.cao@sicnu.edu.cn; 2 School of Mechanical Engineering, Sichuan University, Chengdu 610065, China; scu_wubo@stu.scu.edu.cn (B.W.); 2022323020011@stu.scu.edu.cn (Y.L.); 3 School of Mechanical Engineering, Chengdu University, Chengdu 610106, China; yuanping@cdu.edu.cn; 4 National Engineering Research Center for Biomaterials, College of Biomedical Engineering, Sichuan University, Chengdu 610065, China

**Keywords:** sodium alginate, hydrogels, drug delivery, tissue engineering, biosensing

## Abstract

Sodium alginate, a widely available and high-performance natural polymer, exhibits significant potential for applications in the biomedical field due to its excellent biocompatibility and versatile functionalization capabilities. This review systematically elucidates the fundamental properties and preparation methods of sodium alginate-based hydrogels, analyzing recent advancements in optimizing their mechanical properties, functionalization, and biological characteristics through strategies such as composite material construction, nano-reinforcement, and dynamic crosslinking. Furthermore, it summarizes the multifunctional applications of sodium alginate-based hydrogels in drug delivery, tissue engineering, and biosensing while addressing challenges in practical applications, including insufficient mechanical strength, regulating degradation rates, and maintaining stability in complex biological environments. To overcome these challenges, future research directions are proposed, including performance optimization, intelligent design, novel preparation techniques, and interdisciplinary collaboration, to facilitate the comprehensive transition of sodium alginate hydrogels from laboratory research to clinical applications. This review aims to provide a theoretical foundation and technical support for the fundamental research and biomedical applications of sodium alginate hydrogels while highlighting their promising prospects in addressing complex medical challenges.

## 1. Introduction

With the continuous advancement of biomedical technologies and the growing demand for high-performance biomaterials, natural polymers such as chitosan (CS), hyaluronic acid (HA), sodium alginate (SA), cellulose, gelatin, and xanthan have emerged as a research focus due to their excellent biocompatibility, low toxicity, and biodegradability [1,2]. Among these, SA, a natural polysaccharide extracted from brown algae [3], exhibits significant application potential in the biomedical field due to its unique ionic crosslinking properties, excellent gel-forming ability, and tunable structure [3,4]. SA-based hydrogels, as one of the key forms of sodium alginate, not only possess favorable water retention, flexibility, and tunability [5,6,7] but can also be endowed with diverse new functionalities through chemical modification or compositing with other materials [8,9]. Compared with other natural polymer-based hydrogels, SA-based hydrogels can be rapidly gelled under mild conditions and typically exhibit higher initial wet-state strength than HA or gelatin-based hydrogels. Although HA hydrogels possess favorable viscoelasticity and biocompatibility, their native mechanical strength is limited and they degrade rapidly. Gelatin-based hydrogels, including GelMA, show relatively weak mechanical properties and often require additional crosslinking or composite strategies to enhance their strength [10]. While chitosan complexed with SA can improve stability and mechanical performance through polysaccharide–polyelectrolyte interactions, its properties are strongly influenced by environmental factors such as pH and ionic strength [11]. In summary, SA hydrogels demonstrate outstanding initial strength, formability, and shape retention, making them well-suited for applications requiring high mechanical load-bearing capacity. These properties enable them to meet varied application requirements, leading to their widespread use in fields such as biomedicine [12,13], environmental protection [14,15], and food packaging [16,17].

SA is a linear polysaccharide composed of poly-β-D-mannuronic acid (M) and poly-α-L-guluronic acid (G) units linked via 1,4-glycosidic bonds [18,19]. Its chemical structure, rich in carboxyl and hydroxyl groups [20], enables it to chelate with various divalent or multivalent metal ions (e.g., Ca^2+^, Ba^2+^), rapidly forming a three-dimensional crosslinked network [21]. The ratio of M to G units significantly influences the gelation properties: SA with a high G-unit content tends to form high-strength gels, whereas a higher M-unit content results in greater flexibility [22]. Owing to its three-dimensional crosslinked network, SA-based hydrogels exhibit excellent water absorption and swelling properties. Additionally, these hydrogels demonstrate good biocompatibility. These characteristics make SA an ideal biomedical material, widely applied in drug delivery systems [7], tissue scaffolds [23], and wound healing materials [4]. Furthermore, researchers have expanded its application scope by incorporating nanomaterials [24], designing stimuli-responsive hydrogels, and developing multilayer composite systems [25]. However, despite significant research progress, SA-based hydrogels still face challenges in clinical translation, including insufficient mechanical strength, difficulties in controlling degradation rates, and stability issues in complex biological environments [26]. To address these challenges, researchers have continuously optimized and expanded their functionalities through molecular design, chemical modification, and multi-material compositing [27,28,29], paving the way for further innovative applications in the medical field.

**Scheme 1:**
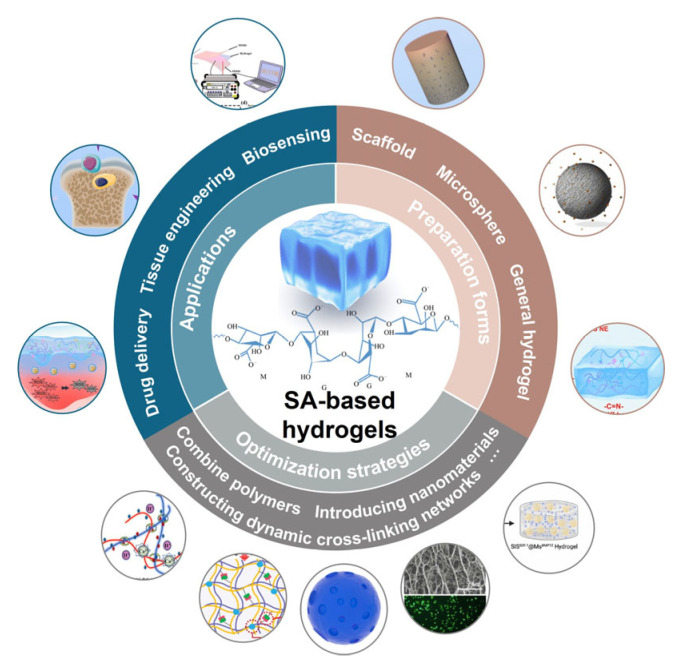
The preparation forms, optimization strategies and applications of SA-based hydrogels.

Based on the above background, this review systematically summarizes the latest research progress on SA-based hydrogels in the field of multifunctional biomedical materials, with a focus on analyzing strategies for performance optimization, functionalization methods, and their applications in drug delivery, tissue engineering, biosensing, and other areas (Scheme 1). Additionally, this paper evaluates the challenges existing in current research and provides an outlook on future development directions, aiming to offer a theoretical foundation and technical reference for fundamental research and clinical applications in related fields.

## 2. Gelation Mechanism and Preparation Methods of SA-Based Hydrogels

### 2.1. Gelation Mechanism

The gelation process of SA-based hydrogels primarily relies on the interactions between crosslinking agents and the molecular chains of SA, forming a three-dimensional network structure. Based on the crosslinking mechanism, gelation methods can be categorized into ionic, covalent, and physical crosslinking.

#### 2.1.1. Ionic Crosslinking

Ionic crosslinking is the primary mechanism for the gelation of SA-based hydrogels. By introducing divalent or multivalent metal ions (e.g., Ca^2+^, Ba^2+^) [30,31], these ions selectively chelate with the carboxyl groups of the G units in the SA molecular chain, forming a crosslinked structure known as the “Egg-Box Model” [21]. This crosslinking method occurs under mild conditions without requiring additional energy, making it suitable for encapsulating temperature-sensitive drugs or bioactive factors. Huang et al. [32] developed chitosan–sodium alginate hydrogels through strong ionic interactions, which, due to their porous internal structure and high hydrophilicity, effectively promoted the proliferation of olfactory ensheathing cells and neural stem cells, significantly enhancing their application potential in neural tissue engineering. Grassi et al. [33] have investigated the performance of alginate/Pluronic (F127) blends for biomedical applications, employing experimental and modeling approaches to study the crosslinking kinetics of alginate chains induced by divalent cations. By measuring the thickness of crosslinked alginate films formed under different exposure times and copper ion (Cu^2+^) concentrations, the kinetic behavior was analyzed. Furthermore, the model parameters, evaluated from the relationship between thickness, Cu^2+^ concentration, and exposure time, were validated through independent experiments. Without introducing additional fitting parameters, the model could predict both the thickness of the hard (crosslinked) layer and the time required for its formation, providing valuable information for stent gel-paving applications.

#### 2.1.2. Covalent Crosslinking

Covalent crosslinking involves the formation of covalent bonds between polymer chains, where functional groups on different polymer segments interact to generate new functional groups [34,35,36]. SA can be oxidized using sodium periodate as the oxidizing agent, which converts the hydroxyl groups at the C2–C3 position into aldehyde groups, thereby enabling subsequent Schiff base reactions with amino groups [37,38]. Ghasemi et al. [39] developed a novel in situ-forming dual-crosslinked hydrogel by exploiting Schiff base chemistry between oxidized sodium alginate (OSA)and silk fibroin (SF), combined with ionic gelation of SA using CaCO_3_. The formation of covalent imine bonds contributed to a significant enhancement in the compressive modulus of the hydrogel. Furthermore, the resulting scaffold demonstrated favorable biocompatibility and considerable potential for promoting osteogenic differentiation. The molecular chains of SA contain abundant active groups such as hydroxyl and carboxyl groups, which can be modified via amide condensation, esterification, and other reactions to introduce small molecular groups capable of crosslinking into hydrogels onto the side chains of sodium alginate. This enables the preparation of covalently crosslinked sodium alginate hydrogels, for example, by introducing methacrylate (MA) groups, phenylboronic acid (BA) groups, thiol (SH) groups, maleimide (MAL) groups, and others [40,41]. Since these modification strategies are widely applied in biomedical engineering, this review will focus on them. Methacrylated SA (SAMA) can be prepared by introducing MA groups onto the hydroxyl groups of sodium alginate, which undergo free-radical polymerization under the action of a photoinitiator and UV light to form covalently crosslinked hydrogel networks [42]. This method allows the fabrication of stable hydrogels with tunable modulus ranging from soft to medium–high, suitable for biomanufacturing and cell encapsulation [43]. By grafting 3-aminophenylboronic acid (PBA) onto the carboxyl groups of sodium alginate, ALG-PBA can be obtained, which crosslinks with polyhydroxyl compounds (e.g., PVA) via reversible boronate ester bonds. This dynamic network is regulated by pH, the pKa of PBA, and diol configuration. Low-pKa PBA enables stable gel formation under physiological pH. The hydrogel possesses self-healing, injectability, and responsiveness to acid, reactive oxygen species, and glucose, making it suitable for drug delivery and soft tissue engineering [44,45]. Through amidation, thiol or maleimide groups can be introduced into the side chains of sodium alginate, followed by thiol-ene click or Michael addition reactions to form thioether bond-crosslinked hydrogels. This system features rapid gelation and good biocompatibility, making it suitable for in situ gel formation and microgel construction [46,47].

#### 2.1.3. Physical Crosslinking

Physical crosslinking primarily relies on hydrogen bonds, hydrophobic interactions, or electrostatic interactions to form gel structures [48,49]. This crosslinking method does not require chemical reagents, making it safer, though the resulting hydrogels often exhibit weaker mechanical properties, rendering them suitable for specific applications. Lang et al. [50] successfully synthesized deferoxamine-grafted sodium alginate (SA-DFA) by adjusting the ratio of SA to deferoxamine mesylate. They found that the aqueous solution could spontaneously form hydrogels due to hydrogen bond interactions, imparting self-healing capabilities with a repair efficiency ranging from 53.64% to 90.16%. Jia et al. [51] significantly improved the mechanical strength and toughness of polyvinyl alcohol (PVA) hydrogels by incorporating an SA nanofiber network. The multiple hydrogen bond interactions effectively dissipated energy, enabling the PVA/SA/NaCl hydrogel to exhibit excellent lubricity and load-bearing performance. Additionally, its bioactivity was confirmed, laying the foundation for the application of novel biomimetic lubricating materials.

#### 2.1.4. Comparison of Three Gelation Mechanism

The mechanical properties of SA-based hydrogels are highly dependent on their gelation mechanisms. And the comparison of the three crosslinking mechanisms is presented in Table 1. Hydrogels formed through physical crosslinking primarily rely on hydrogen bonds and chain entanglements, which result in relatively poor mechanical strength, stiffness, and toughness; however, they exhibit good self-healing capability and reversibility [52,53]. Ionic crosslinking, as the most established method, offers the key advantage of highly tunable mechanical properties—such as strength and modulus—along with energy dissipation capacity and moderate self-healing behavior endowed by the reversible breakage and reformation of ionic bonds [54,55]. Nevertheless, its long-term stability in physiological environments remains a challenge [56]. In contrast, covalent crosslinking utilizes chemical crosslinkers to form stable covalent bonds between molecular chains, constructing a robust and permanent three-dimensional network [57,58]. Its most prominent features include superior mechanical strength, stiffness, and excellent chemical stability surpassing those of the other two methods, though these are often accompanied by brittle fracture and a complete lack of self-healing ability [59]. It is important to note that this does not apply to dynamic reversible covalent crosslinking, such as boronate ester and Schiff base reactions, which uniquely combines the robustness of covalent bonds with inherent self-healing properties [60,61].

The selection of the crosslinking mechanism essentially represents a trade-off among mechanical properties, stability, and dynamic functionality. Ionic crosslinking has become a mainstream choice for biomedical applications due to its favorable biocompatibility and tunability. In scenarios where extreme mechanical strength and long-term stability are required, covalent crosslinking or a hybrid approach combining it with ionic crosslinking is more suitable. Conversely, physical gelation is appropriate for applications that prioritize reversible responsiveness over high mechanical performance. In practical research, hybrid crosslinking strategies are frequently employed to leverage the advantages of each method, thereby enabling the fabrication of composite hydrogels that exhibit both excellent bioactivity and superior mechanical properties.

### 2.2. Preparation Forms of SA-Based Hydrogels

To meet diverse application requirements, the preparation forms of SA-based hydrogels have been continuously developed and optimized. The following are several common preparation forms:

#### 2.2.1. Traditional SA-Based Hydrogels

Traditional hydrogels are prepared by dripping an SA solution into a solution containing a crosslinking agent (e.g., CaCl_2_), rapidly forming gels or gel films. This method is simple to operate and suitable for producing materials in forms such as hydrogels, hydrogel fibers, or films, which are widely used in drug delivery and tissue engineering. For instance, Li et al. [62] prepared pH-responsive hydrogels based on soybean protein nanofibers (SNF) and SA through simple ionic crosslinking to meet the application requirements of hydrogels in different environments. He et al. [63] constructed peptide-polysaccharide hybrid hydrogels with excellent stability and drug delivery performance by co-assembling Fmoc-FF peptides with alginate triggered by calcium ions. The synergistic effect of non-covalent and ionic interactions enabled the hydrogel to exhibit enhanced stability in water and phosphate buffer solutions, while sustained and controlled drug release was achieved by adjusting the concentration ratio of Fmoc-FF peptides to alginate. Han et al. [64] successfully prepared ionically crosslinked alginate-hyaluronic acid hydrogels with reversible dynamic metal-ligand interactions by simply assembling a mixture of alginate and hyaluronic acid with Fe^3+^ complexes. These hydrogels rapidly recovered after damage, exhibited shear-thinning behavior, and demonstrated favorable injectability. The sustained release of iron ions due to local degradation endowed the hydrogels with potential long-term antibacterial activity against various bacteria.

#### 2.2.2. SA Hydrogel Microspheres

SA hydrogel microspheres are prepared by dispersing sodium alginate solution into microdroplets via ion spraying or emulsification techniques, followed by contact with a crosslinking agent to form microspheres. This method produces microspheres with stable and uniform particle sizes and short gelation times, effectively preventing the deactivation of active substances within the gel microspheres [65,66]. These microspheres are commonly used in controlled drug release and cell encapsulation systems. Additionally, the preparation of sodium alginate microspheres (SAMs) is conducted under mild conditions, enabling large-scale production. Luo et al. [67] prepared two types of pH-dependent SA hydrogel microspheres using CaCl_2_ and FeCl_3_ as crosslinking agents, significantly enhancing their stability in gastric environments. This approach effectively protected curcumin and extended its release time, thereby opening new prospects for the oral delivery of low-density lipoprotein-based nanogels. Wang et al. [68] successfully developed CS-PA/calcium alginate/SB (CPC/SB) microspheres using a microemulsion method combined with CS, protocatechuic aldehyde (PA), SA, and sodium butyrate (SB) to protect the intestinal barrier from damage caused by inflammatory bowel disease (IBD) and severe acute pancreatitis (SAP). Experimental results demonstrated that CPC/SB microspheres exhibited high histocompatibility and significant antioxidant capacity, with no notable impact on cell viability or migration in vitro. Microfluidic technology has provided innovative tools for preparing alginate hydrogel microspheres with diverse morphologies, structures, and compositions [65,69]. Liao et al. [70] successfully fabricated live alginate hydrogel microspheres loaded with photosynthetic autotrophic microalgae using microfluidic electrospray technology. This innovative method not only ensured high biocompatibility and efficient oxygen release but also significantly promoted collagen deposition and angiogenesis, providing robust support for wound healing. Wang et al. [71] developed sodium alginate microspheres (SAMs) containing M2 macrophage membrane (M2M)-coated Janus nanomotors (referred to as Motor@M2M@SAM) via microfluidic techniques to stably maintain the functional proteins of M2 macrophages (M2Ms) through the gastrointestinal tract for targeted treatment of ulcerative colitis. Experimental results showed that Motor@M2M@SAM significantly alleviated inflammation and improved pathological conditions in inflamed colons, representing a promising therapeutic strategy for ulcerative colitis. In terms of controlled release systems, microencapsulation technology has emerged as a research hotspot in recent years [72,73]. Conventional and uniform microcapsule structures can be formed by introducing techniques such as emulsification [74], spray drying [75] or microfluidic packaging planning [76] during the preparation process. Such microcapsules effectively protect loaded agents from environmental influences and enable long-term, controllable drug release by regulating wall thickness, degradation rate, and pore characteristics [77]. Studies have shown that this strategy not only prolongs drug action in vivo but also enhances therapeutic efficacy, making it particularly suitable for oral formulations, localized delivery, and cell encapsulation applications [78,79].

#### 2.2.3. 3D-Printed SA Hydrogel Scaffolds

3D printing technology enables the preparation of hydrogel scaffolds with complex shapes and precise structures by layer-by-layer deposition of SA solution, followed by reaction with a crosslinking agent [80]. This approach holds significant application potential in personalized medicine, particularly in bone tissue engineering [81] and soft tissue repair. Chen et al. [82] demonstrated that alginate hydrogel constructs prepared using a 3D bioprinting system combined with an immersion crosslinking process, by controlling the initial crosslinking density, effectively supported cell viability and cartilage extracellular matrix deposition in cartilage tissue engineering, thus providing new application potential in this field. Although SA hydrogels are a promising bioink source for 3D bioprinting due to their low cost, excellent biocompatibility, and rapid ionic gelation properties, their key limitations include the lack of cell adhesion groups [83], unstable mechanical properties [84], and poor printability [85]. These limitations have driven the need for improvements in SA-based bioinks, thereby promoting the development of advanced material formulations and biomanufacturing strategies. Wang et al. [86] showed that borate bioactive glass (BBG), particularly when combined with SA, enhances the precision and biocompatibility of 3D bioprinting. By controlling the release of calcium ions, BBG plays a critical role in improving the self-gelation of SA, making it a promising material for complex bioprinting applications, including bone and soft tissue implants. Shu et al. [87] proposed a three-stage crosslinking process for alginate hydrogels: initial calcium ion crosslinking to achieve printability, secondary calcium ion crosslinking to enhance the rigidity of printed alginate hydrogels, and tertiary barium ion crosslinking to ensure long-term stability in culture media, enabling the bioprinting of more complex alginate hydrogel structures. Naji et al. [88] constructed urethral epithelial scaffolds using 3D printing technology, with mechanical properties positively correlated with increased alginate content. These scaffolds significantly improved the survival rate and functional gene expression of bladder smooth muscle cells, providing a promising tissue engineering approach for urethral epithelial reconstruction.

Cryogenic (freezing) printing technology is also noteworthy. By rapidly freezing deposited filaments to lock their geometry and utilizing ice crystals as templates, cryogenic printing enables the creation of anisotropically ordered or graded channels, thereby achieving high shape fidelity and strong interlayer bonding [89,90]. This process also allows for the design of controlled multiscale interconnected porous architectures and subsequent secondary cross-linking for mechanical reinforcement [91]. Moreover, it preserves cell viability during printing and controlled thawing while supporting scalable manufacturing. Cryogenic printing can systematically improve the mechanical stability, morphological precision, and pore structure engineering of hydrogel scaffolds [92,93], providing a reproducible and scalable manufacturing pathway for personalized tissue-engineered constructs. For instance, Luo et al. [94] incorporated α-tricalcium phosphate powder into SA hydrogel containing FePSe_3_-nanosheets and constructed a multifunctional therapeutic platform (TCP-FePSe_3_) for synergistic osteosarcoma treatment via cryogenic 3D printing. This technique eliminates the need for a sintering process and enables the efficient loading of bioactive FePSe_3_ nanosheets within the scaffold. The resulting TCP-FePSe_3_ composite scaffolds feature a highly porous structure and ordered surface topography, which facilitate stem cell recruitment and neovascularization. These properties further promote mechanical interlocking between the implant and surrounding native bone, demonstrating significant potential for inhibiting postoperative osteosarcoma recurrence and supporting subsequent vascularized bone regeneration.

In conclusion, with the rapid advancement of modern manufacturing strategies, the controllability and scalability of sodium alginate hydrogels have been significantly improved. Microfluidic technology allows precise manipulation of single-droplet gelation processes, yielding microspheres or microcapsules with adjustable sizes and uniform structures. Meanwhile, 3D printing and cryogenic printing techniques enable the accurate fabrication of customized scaffolds with complex architectures and hierarchical porosity, while preserving cell viability and achieving controlled multi-material integration. Combining these advanced processes, hydrogels now exhibit enhanced mechanical properties and biocompatibility, while also meeting clinical demands for large-scale, standardized, and personalized production. Overall, these systematic advances in gelation mechanisms, controlled release functionality, and manufacturing processes have laid a more solid foundation for the clinical translation of sodium alginate hydrogels in tissue engineering, regenerative medicine, and drug delivery.

## 3. Performance Optimization Strategies for SA-Based Hydrogels

Despite the significant application potential of SA-based hydrogels in the biomedical field, their weak mechanical properties, limited functionality, and stability issues in complex biological environments restrict their broader applications. Consequently, modification and functional enhancement through performance optimization strategies have become a key focus of current research. The following sections discuss performance optimization strategies for SA-based hydrogels from three perspectives: mechanical property enhancement, functionalization modification, and degradation rate regulation (Table 2). The integrated application of these optimization methods will further expand the prospects of SA-based hydrogels as multifunctional biomaterials.

### 3.1. Mechanical Property Enhancement

#### 3.1.1. Combining Polymers

**Figure 1:**
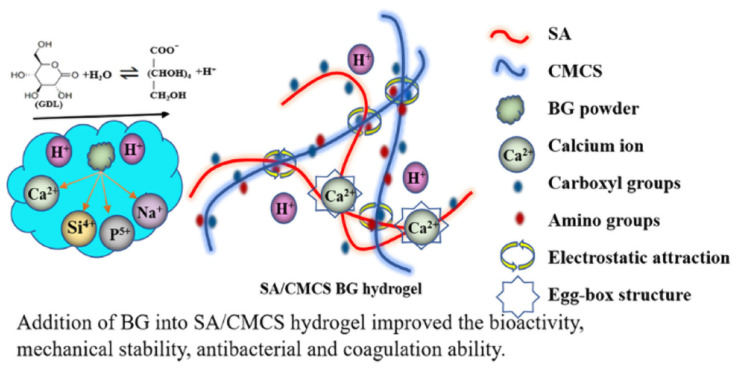
The gelation mechanism of SA/CMCS/BG hydrogels. Copyright Permission from Li et al. [107] ACS Applied Bio Materials 2021.

The combination of SA with other natural or synthetic polymers is a common strategy to enhance its mechanical properties. For instance, compositing SA with materials such as chitosan [32], gelatin [105], or PEG [106] can significantly improve its strength, elasticity, and toughness. The cationic properties of chitosan can form a more stable crosslinking network with the anionic properties of SA, while the bioactivity of gelatin enhances cell adhesion. By optimizing the composition ratios and crosslinking conditions, a balance between mechanical properties and biological functionality can be achieved. For example, Li et al. [12] developed an injectable self-healing hydrogel containing islet-integrated microfiber scaffolds for islet transplantation. The self-healing matrix, composed of OSA and CMCS, formed dynamic Schiff base bonds between the aldehyde groups of OSA and the amino groups of CMCS, facilitating reversible crosslinking and enabling the hydrogel’s formation and inherent self-healing capability. Bushra et al. [95] prepared magnetic nanocellulose via co-precipitation and incorporated it into SA hydrogel beads, significantly enhancing the mechanical strength and enabling controlled drug release, thereby improving drug delivery performance. Li et al. [107] incorporated BG into SA/CMCS hydrogels, markedly improving mechanical stability and bioactivity, which accelerated skin wound healing, making SA/CMCS/BG hydrogels an ideal candidate for clinical wound dressings (Figure 1). Zhang et al. [108] diffused high-molecular-weight chitosan into PVA/SA (PS) hydrogels, successfully preparing a PSCS double-network hydrogel through physical crosslinking without using toxic chemical crosslinking agents. This hydrogel exhibited excellent swelling and mechanical properties, significantly enhancing its potential for applications under stimuli-responsive conditions, making it a promising material for smart actuators or soft robotics. Nevertheless, this strategy offers limited enhancement in mechanical strength, exhibiting an inherent performance ceiling, while multicomponent systems may face challenges such as phase separation and batch-to-batch variability. Hence, it is particularly suitable for applications where absolute strength is not critical, but a balanced overall performance and biological functionality are emphasized—such as in tissue engineering scaffolds, wound dressings, and drug delivery systems.

#### 3.1.2. Reinforcement Nanomaterials

**Figure 2:**
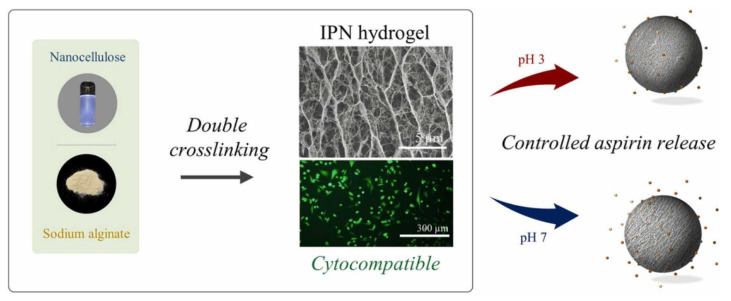
Schematic diagram of the preparation of TOCN/SA interpenetrating network hydrogels and their PH sensitivity. Copyright Permission from Fan et al. [111] Industrial Crops and Products 2023.

The incorporation of nanomaterials provides new avenues for optimizing the mechanical properties of SA-based hydrogels. For instance, adding nanomaterials such as nanocellulose, graphene oxide (GO), silica nanoparticles, or hydroxyapatite (HA) can significantly enhance the rigidity, compressive strength, and wear resistance of hydrogels. The core advantage of this strategy lies in its exceptionally high reinforcement efficiency, where even minimal incorporation can lead to a drastic improvement in mechanical properties, while simultaneously conferring additional functionalities such as electrical conductivity, thermal conductivity, or antibacterial activity to the hydrogel. Lu et al. [26] developed a stable hydrogel drug carrier with excellent biocompatibility, biodegradability, and low toxicity by incorporating CNF and hydrothermally prepared carbon microspheres into a hydrogel network formed by chelation of SA with Ca^2+^, thereby improving the mechanical and degradation properties of the drug carrier. Bhandari et al. [96] demonstrated that compositing CNC with SA significantly enhanced the physical properties and stability of the composite hydrogel, effectively reducing the diffusion rate of a water-soluble polyphenol model, GA, thus offering broad application prospects in bioencapsulation. Additionally, these nanomaterials can impart new properties to hydrogels, such as conductivity [109] and stimuli-responsiveness [110], expanding their applications in biosensing and smart materials. Fan et al. [111] found that an interpenetrating network hydrogel formed by nanocellulose and SA, through an acetic acid coagulation bath and Ca^2+^ chelation, significantly extended the release time of aspirin to approximately 60 h due to dual crosslinking effects. This enriched the hydrogel’s network structure, making pH-sensitive nanocellulose-sodium alginate interpenetrating network hydrogels an ideal carrier for long-term aspirin release (Figure 2). Furthermore, by optimizing the composition of nanocellulose-alginate hydrogels and introducing biofunctionalization, multiple performance aspects in 3D printing can be enhanced, significantly improving their application potential in biomedical devices, wearable sensors, and drug delivery materials [112].

In recent years, the incorporation of photoluminescent nanomaterials into SA hydrogels has garnered significant attention to substantially broaden their functional applications. The integration of such nanomaterials—including quantum dots (QDs) [113,114], upconversion nanoparticles (UCNPs) [115,116], and fluorescent carbon dots(CDs) [117,118]—imparts luminescent properties to otherwise transparent or optically inert hydrogels, thereby enabling real-time, high-resolution visualization and monitoring of hydrogel structures via non-invasive optical techniques. Lu et al. [119] developed a pH-responsive, biodegradable hydrogel soft actuator based on CDs and SA that synchronously exhibits color modulation and shape morphing. The incorporation of CDs not only endows the hydrogel with pH-tunable fluorescence but also reinforces its mechanical properties through covalent and hydrogen-bond cross-linking, enabling synergistic chromatic–mechanical responses and advancing its utility in intelligent soft robotics. Tahmasebi and Mohammadi [120] fabricated a pH-responsive hydrogel based on okra polysaccharide and SA. By integrating silver nanoparticles and carbon quantum dots (CQDs), the system enables targeted drug delivery for colorectal cancer and exhibits multifunctional properties, including antibacterial, antioxidant, and photoluminescent activities, highlighting its promising biomedical applications. The incorporation of CQDs not only endows the hydrogel with photoluminescent properties for real-time tracking but also enhances drug loading and release control through surface functional groups. Moreover, CQDs synergistically improve antioxidant and antibacterial capacities while reinforcing structural stability, making them a critical component in constructing intelligent and multifunctional drug delivery systems.

However, the drawbacks of this strategy are equally pronounced: nanoparticles are highly prone to aggregation, which can introduce structural defects and compromise the reinforcement effect. Moreover, the biosafety and long-term toxicity of certain nanomaterials remain poorly understood, raising concerns for clinical translation. This strategy is primarily employed in applications demanding stringent mechanical performance or specialized functionalities, such as high-load bone regeneration, flexible electronic sensors, and wearable devices.

#### 3.1.3. Constructing Dynamic Cross-Linking Networks

**Figure 3:**
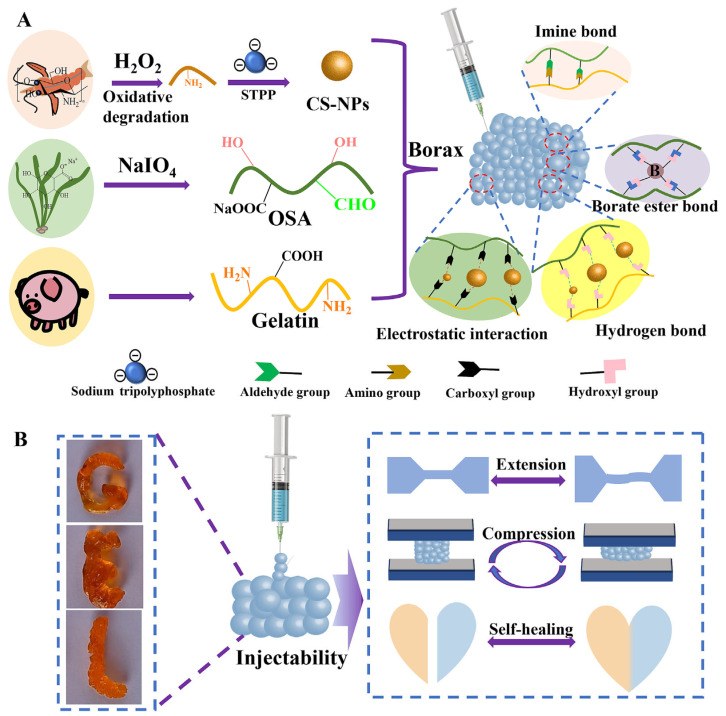
Schematic diagram of the preparation of CS-NPs@OSA-l-Gtn hydrogel (**A**) and its properties (**B**). Copyright Permission from Ito et al. [125] Carbohydrate Polymers 2024.

By introducing dynamic crosslinking mechanisms, such as reversible dynamic covalent bonds or physical interactions, hydrogels can achieve enhanced strength while gaining self-healing capabilities [121,122]. For instance, dynamic covalent bonds like boronate ester bonds or Schiff base bonds, can preferentially dissociate under external stress to dissipate energy and subsequently recombine after unloading, thereby endowing the hydrogels with remarkable toughness, fatigue resistance, and self-healing capabilities [123,124]. Ito et al. [125] incorporated chitosan nanoparticles (CS-NPs) into the dynamic polymer networks of OSA and gelatin (Gtn) in the presence of borax and developed an injectable nanocomposite hydrogel (CS-NPs@OSA-l-Gtn). This network comprises dynamic covalent bonds (imine and boronate ester bonds) and non-covalent interactions (electrostatic forces and hydrogen bonds), which work synergistically to endow the hydrogel with self-healing, injectability, and enhanced mechanical properties, while also enabling the sustained release of bioactive molecules. This design strategy offers a novel approach for developing high-strength, functionalized natural polymer-based hydrogels suitable for biomedical applications (Figure 3). Furthermore, by modulating the rate of dynamic crosslinking, precise control over the mechanical and degradation properties of hydrogels can be achieved [126]. Zhou et al. [127] developed a biomimetic hydrogel to improve the osteogenic microenvironment and promote stem cell homing. The research team covalently modified SA with Ca^2+^ to adjust its stiffness, enhancing the paracrine properties of bone marrow mesenchymal stem cells (BMSCs), which promoted coupled osteogenesis and angiogenesis while exhibiting immunomodulatory functions. Zou et al. [97] demonstrated that by constructing a covalent network hydrogel, OSA not only crosslinked with chitosan to form a hydrogel network but also connected with a PAM network through Schiff base bonds and hydrogen bonds, endowing the hydrogel with excellent toughness and self-healing properties. This hydrogel retained 40% fracture stress and 53% fracture strain even when stretched to 20 times its original length. Tsai et al. [128] utilized poly(lactic-co-glycolic acid) (PLGA) as a template to regulate the conformation of calcium-ion-crosslinked alginate, successfully developing a novel reversible and smart interpenetrating polymer network (IPN) hydrogel. This hydrogel could absorb large amounts of wound exudate, maintain shape stability, and facilitate stable loading and controlled release of vascular endothelial growth factor (VEGF), thereby providing a promising delivery platform for accelerating wound healing. However, the chemical modifications involved are often synthetically challenging, and the reversible nature of the dynamic bonds may lead to creep under sustained loads, making high absolute strength a relative weakness. This approach is particularly suited for applications requiring dynamic mechanical properties, such as injectable hydrogels, self-healing devices, soft robotics, and dynamic cell culture models.

### 3.2. Functionalization Modification

#### 3.2.1. Intelligent Responsive Modification

**Figure 4:**
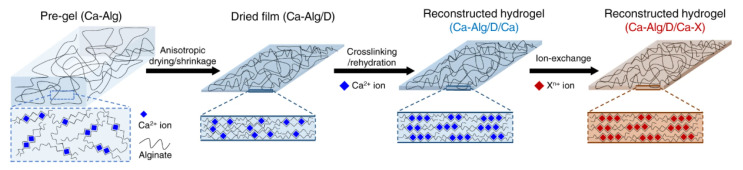
Schematic diagram of reconstruction of SA hydrogels. Copyright Permission from Kim et al. [131] Nature Communications 2022.

Smart responsive hydrogels represent a key direction in the functionalization of SA-based hydrogels. By incorporating molecules or materials sensitive to external stimuli, hydrogels can be endowed with responsiveness to pH, temperature, light, electric fields, or magnetic fields [129,130]. For instance, the incorporation of temperature-sensitive polymers, such as poly(N-isopropylacrylamide) (PNIPAM), enables hydrogels to undergo volume expansion and contraction at different temperatures, while compositing with magnetic nanoparticles yields smart hydrogels responsive to magnetic fields. Such functionalization modifications significantly expand the applications of SA-based hydrogels in drug delivery and biosensing. Abdi et al. [98] successfully prepared novel antibacterial magnetic/pH-sensitive hydrogel beads. The incorporation of Ag and Fe_3_O_4_ nanoparticles reduced the swelling capacity and imparted pH-dependent swelling behavior, resulting in pronounced drug release behavior under simulated physiological pH conditions, particularly at pH 7.4, where the drug release rate reached approximately 83% after 200 min. Additionally, these hydrogel beads exhibited controlled drug release under magnetic fields and demonstrated strong antibacterial activity against Staphylococcus aureus and Escherichia coli, offering significant potential for drug delivery systems. Kim et al. [131] developed ultra-strong, ultra-tough, and conductive SA hydrogels through a simple reconstruction method involving anisotropic drying and shrinkage of pre-gels, followed by ionic crosslinking and rehydration in an ionic solution to form a tightly interconnected network structure (Figure 4). The resulting hydrogels exhibited tensile strength and elastic modulus ranges of 8–57 MPa and 94–1290 MPa, respectively, while accommodating sufficient cations to achieve high ionic conductivity. By incorporating the conductive polymer PEDOT: PSS, ionic/electronic conductive hydrogels were fabricated, and a three-layer hydrogel supercapacitor using these hydrogels as electrolytes demonstrated excellent electrochemical stability, providing a new direction for the development of functional hydrogels. Xue et al. [99] proposed an EPC-SA hydrogel prepared by covalently coupling SA to temperature-sensitive polyurethane, which responded to both temperature and Ca^2+^ stimuli, forming an orthogonal crosslinking structure. This significantly enhanced the mechanical strength and enabled tunable gel strength, demonstrating substantial potential in cell-based biomedical applications, such as cell encapsulation and cell therapy.

Four-dimensional (4D) printing integrates conventional 3D printing with “smart materials”, enabling 3D-printed structures to undergo programmed morphological changes over time in response to external stimuli [132,133]. As an emerging technology, 4D printing has expanded the application prospects of stimulus-responsive hydrogels. Zhang et al. [134] fabricated a 4D-printed SAMA hydrogel that allows precise programming of shape transformation, facilitating the fabrication of intricate miniature structures—such as microvascular scaffolds—that are challenging to produce using traditional methods. After multi-crosslinking, the hydrogel exhibited significantly enhanced mechanical properties along with good biocompatibility, offering a novel strategy for applications in soft robotics and biomedical devices. Huang et al. [135] developed a novel self-bending hydrogel based on SAGMA (sodium alginate modified with glycidyl methacrylate) loaded with nanospherical mineralized collagen, which was subsequently fabricated via 4D printing. This 4D-printed SAGMA hydrogel scaffold demonstrates integrated advantages including autonomous bending, tunable curvature, high mechanical strength, excellent biocompatibility, and strong osteogenic promotion capability. It is particularly suitable for repairing irregular bone defects with complex curvatures, such as cranial defects, thereby providing an intelligent solution for bone tissue engineering.

#### 3.2.2. Surface Functionalization

**Figure 5:**
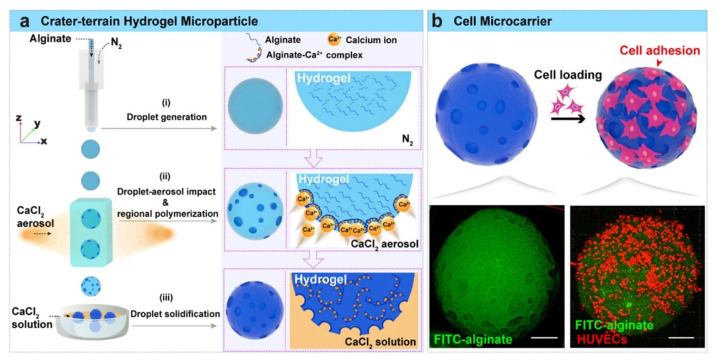
(**a**) The preparation of CTMs using microfluidic droplet technology. (**b**) Schematic diagram of CTMs for cell microcarriers. Copyright Permission from Lin et al. [136] ACS Applied Materials & Interfaces 2023.

Surface functionalization of SA-based hydrogels through chemical modification can significantly enhance their bioactivity. For instance, introducing cell adhesion peptides (e.g., RGD sequences) or growth factors (e.g., VEGF, TGF-β) on the hydrogel surface can improve cell adhesion, proliferation, and differentiation. Leach et al. [100] modified SA hydrogels with VEGF-mimetic peptides (QK) to enhance the secretion of pro-angiogenic factors and incorporated RGD to promote cell adhesion, while controlling osteogenic differentiation by adjusting the viscoelasticity of the hydrogels. In vivo experiments demonstrated that this approach promoted vascular and bone regeneration in rat cranial defects within 12 weeks. These findings indicate that such methods can simultaneously enhance the regenerative potential of mesenchymal stem cells (MSCs) in multiple aspects and improve neovascularization in tissue engineering. Additionally, surface functionalization can impart properties such as antibacterial and anti-inflammatory activities, thereby increasing the application value of hydrogels in wound healing and implant materials. Tran et al. [101] developed a thermosensitive hydrogel system by copolymerizing amidinated SA with poloxamer F127, which not only endowed the hydrogel with excellent antibacterial properties but also exhibited good biocompatibility and cell culture capabilities in physiological media, providing a novel cell delivery platform for various tissue engineering applications. Lin et al. [136] utilized microfluidic droplet technology to design hydrogel particles with a “crater-terrain” surface structure (Crater-terrain microparticles, CTMs). The rough surface of these CTMs induced spontaneous protein adsorption, forming a protein corona binding layer on the particle surface. Through interactions between the surface proteins and cell membrane receptors, these particles guided cell adhesion to their surface (Figure 5). These particles exhibited superior cell affinity, making them highly suitable as next-generation cell carriers for cell biology research.

#### 3.2.3. Drug Loading and Controlled Release Design

**Figure 6:**
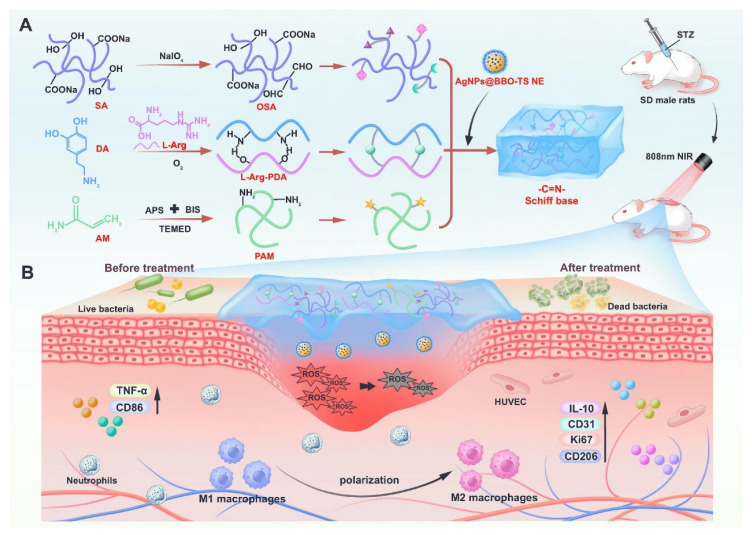
(**A**) Schematic diagram of the preparation for LPOA hydrogels. (**B**) The role of LPOA hydrogels in treating diabetic wounds. Copyright Permission from Pang et al. [123] Chemical Engineering Journal 2025.

Functionalization modification can also achieve efficient drug loading and precise controlled release by adjusting the internal structure and pore distribution of hydrogels [26,137,138,139]. Popov et al. [140] found that embedding callus tissue cells into SA hydrogels significantly reduced porosity, enhanced gel strength and thermal stability, and improved the encapsulation efficiency and release rate of grape seed extract (GSE), thereby providing an ideal carrier with physicochemical and textural properties for colon drug delivery systems. In the context of articular cartilage regeneration, traditional hydrogels with nanoscale pores often lead to poor cell and tissue infiltration. To address this, Li et al. [102] designed and prepared a novel injectable macroporous hydrogel system (SA/HAexo-PLGAKGN), composed of SA/HA microfibers loaded with specific exosomes and PLGAKGN microspheres, to sequentially deliver bioactive substances to various stages of cartilage regeneration. The macroporous SA/HAexo-PLGAKGN hydrogel not only significantly improved tissue infiltration into the hydrogel but also enabled sequential delivery of loaded bioactive substances, enhancing cartilage regeneration in a rat cartilage defect model. Single SA hydrogels typically have large pores and poor stability, whereas the construction of dual-crosslinked network hydrogels helps optimize their physicochemical properties and improve drug delivery efficacy [141]. Pang et al. [123] utilized the basic properties of L-arginine to induce dopamine self-polymerization, followed by crosslinking with OSA via Schiff base reactions, combined with PAM to form a dual-network system. They embedded AgNPs@BBO-TS NE to enhance antibacterial activity, successfully developing a multifunctional LPOA hydrogel with excellent skin adaptability, self-healing capability, and mechanical toughness, capable of withstanding high-frequency mechanical stress in dynamic wounds (Figure 6). Li et al. [103] constructed a dual-network alginate hydrogel (siHPTs@ZA) for rheumatoid arthritis (RA) treatment by crosslinking SA with Zn^2+^ and further crosslinking with hyperbranched poly(β-amino ester) (HB-PBAEs), loaded with TNF-α siRNA (siHPTs). Experimental results demonstrated that this hydrogel could reprogram the immune-metabolic microenvironment in RA, reducing hypoxia and glycolysis while enhancing the FAO pathway and M2 macrophage polarization, making it a promising platform for next-generation high-efficiency RA treatment.

### 3.3. Regulating Degradability

**Figure 7:**
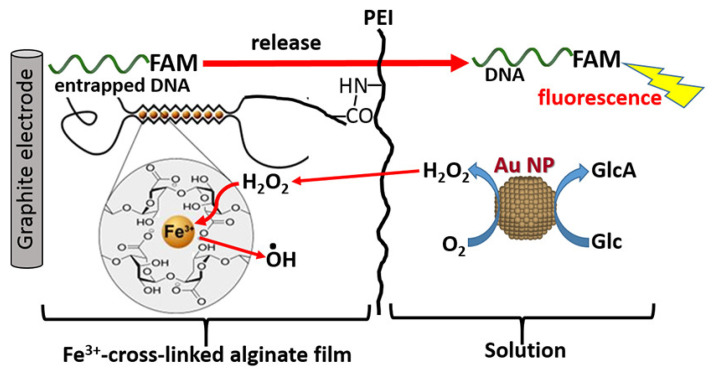
Schematic diagram of the DNA-FAM release from the Fe^3+^-cross-linked alginate hydrogel stimulated with H2O2 added to the bulk solution. Copyright Permission from Katz et al. [145] ACS Applied Bio Materials 2020.

The degradation properties of SA-based hydrogels are critical for their biomedical applications, and the degradation rate can be significantly influenced by adjusting the type and concentration of crosslinking agents [142]. Accelerating the degradation rate of hydrogels can enhance tissue infiltration. Li et al. [104] proposed a novel strategy to regulate the degradation of SA hydrogels by grafting DFO onto SA via an amide reaction, preparing injectable DFO-SA/BG (G-DFO-SA/BG) hydrogels. The grafting of DFO reduces the crosslinking density of SA/BG hydrogels by consuming some carboxyl groups on SA, enabling the preparation of rapidly degradable G-DFO-SA/BG hydrogels without compromising their biocompatibility. By day 14, the G-DFO-SA/BG hydrogel retained only 48% of its initial mass, whereas both SA/BG and F-DFO-SA/BG remained above 100% because of continuous swelling. Over the same 5-day immersion, the compressive modulus of G-DFO-SA/BG dropped from 0.06 MPa to 0.01 MPa, corresponding to an 83% loss that was markedly greater than the 75% decline observed for SA/BG or the 50% loss recorded for F-DFO-SA/BG. Experimental results demonstrated that DFO-SA/BG (G-DFO-SA/BG) hydrogels exhibited faster degradation rates and enhanced tissue infiltration, inducing greater vascular formation and significantly accelerating the wound healing process, thus showing broad application potential in tissue regeneration. In drug delivery applications, designing more stable hydrogels with slower degradation rates is often necessary to protect drugs and achieve targeted release [143]. Ge et al. [144] prepared a novel composite hydrogel by crosslinking collagen with SA under the action of Ca^2+^. This hydrogel exhibited higher water content, greater mechanical strength, slower collagen degradation rates (approximately 60% of the original mass remained after 12 h of incubation with type-I collagenase, indicating a markedly reduced degradation rate and a pronounced sustained-release profile), and low cytotoxicity, significantly improving wound healing efficiency and providing a new material option for treating chronic wounds. Furthermore, the introduction of enzymatically degradable polymers enables controlled degradation of hydrogels in vivo. Katz et al. [145] prepared calcium alginate hydrogel films with varying compositions using electrochemical methods and investigated their interfacial reactions to achieve biomolecule release. Experimental results showed that using Au nanoparticles as nanozyme catalysts could trigger the degradation/dissolution of Fe^3+^-cross-linked calcium alginate hydrogel films in response to glucose signals, effectively addressing the uncontrolled leakage of DNA molecules from the gel. This enabled signal-triggered DNA release in various configurations, ranging from simple addition of H_2_O_2_ to the solution to in situ generation of H_2_O_2_ within the calcium alginate films by nanozymes, significantly enhancing the versatility of nanozymes in signal-controlled biomolecule release systems (Figure 7).

## 4. Multifunctional Applications of SA-Based Hydrogels

Based on their application forms and functional characteristics in specific scenarios, the multifunctional applications of SA-based hydrogels can be categorized into drug delivery, tissue engineering and regenerative medicine, and biosensing and diagnostics. To provide clearer guidance for the selection of optimization strategies in practical applications, we have summarized the correspondence between the performance optimization strategies discussed in Section 3 and the specific application areas in Table 3. This comparative analysis highlights how different modification approaches can be tailored to meet the demands of diverse biomedical applications, thereby enhancing the design rationality and functional precision of SA-based hydrogels.

As illustrated in Table 3, the optimization strategies are highly application-specific. For instance, intelligent responsive modification and nanomaterial reinforcement are particularly suitable for drug delivery and biosensing, where stimuli-triggered behavior and enhanced electrical/optical properties are critical. In contrast, combining polymers and surface functionalization are more commonly employed in tissue engineering to improve mechanical properties and cellular interactions. Furthermore, degradation regulation plays a vital role in regenerative medicine by matching the hydrogel’s resorption rate with tissue regeneration timelines.

The following subsections will elaborate on each application area, with a focus on how these optimization strategies have been implemented to address specific biomedical challenges.

### 4.1. Drug Delivery

SA-based hydrogels, through various functional designs, have emerged as versatile platforms for intelligent drug delivery, which can be primarily categorized into the following three strategies.

The controlled release mechanism primarily relies on the intrinsic physicochemical properties of hydrogels, such as swelling-contraction behavior and ionic crosslinking degradation, forming the fundamental basis for constructing stimuli-responsive delivery systems. SA-based hydrogels can achieve sustained drug release through swelling-contraction behavior and the degradation process of ionic crosslinking. For instance, the swelling properties of hydrogels enable stable drug release over a specific period, while adjusting the crosslinking agent ratio to control hydrogel porosity significantly affects drug release behavior. The introduction of porous structures accelerates drug release rates, whereas non-porous hydrogels result in slower drug release, enabling precise control over drug release behavior [149]. Studies have demonstrated that SA hydrogels exhibit excellent performance in the controlled release of anticancer drugs [13] and anti-inflammatory drugs [150]. Thach et al. [151] showed that incorporating polydopamine into calcium alginate crosslinked hydrogels significantly enhanced water absorption, swelling performance, and drug loading capacity, thereby accelerating drug release rates.

Active targeting strategies represent a crucial development direction in precision medicine, significantly enhancing therapeutic efficiency while reducing systemic side effects through precise drug delivery to lesion sites. Combining SA hydrogels with magnetic nanoparticles or surface-modified targeting ligands enables targeted drug delivery. For example, Yu et al. [152] utilized constant β-D-mannuronic acid(M) units and modulated α-L-guluronic acid (G) units in the alginate (ALG) structure to construct a multifunctional nanoplatform, biotinylated aldehyde alginate-doxorubicin nano micelle (BEA-C=N-DOX-M), for synergistic chemo-immunotherapy. Specifically, vitamin Bio, with high tumor-targeting specificity, and the broad-spectrum antitumor drug doxorubicin (DOX), which induces immunogenic cell death (ICD), were conjugated to the reactive G units of oxidized alginate via ester bonds and acid-sensitive imine bonds, respectively, as targeting and therapeutic components. Liu et al. [153] functionalized halloysite nanotubes (HNTs) with tea polyphenol (EGCG), leveraging its excellent adhesion and metal coordination properties, yielding MPN@HNTs, which were blended with SA solution to prepare a hydrogel microsphere system (MHBSA) for probiotic delivery and targeted treatment of inflammatory bowel disease (IBD). The HNTs targeted inflamed colon sites via electrostatic interactions, with their rod-like microstructure prolonging mucosal retention time. MPN@HNTs scavenged reactive oxygen species (ROS), enhanced probiotic antioxidant capacity, and modulated gut microbiota, providing a novel approach for IBD treatment. Warkar et al. [154] successfully prepared novel hydrogel microspheres based on tragacanth gum (TG), β-cyclodextrin (β-CD), and SA through ionic crosslinking, improving the targeted delivery of the hydrophobic drug aspirin. Experimental results showed that TG/β-CD/SA microspheres exhibited high drug loading (19%) and encapsulation efficiency (63.33%), with a higher drug release rate (59.9%) at pH 7.4, demonstrating significant potential as a delivery system for hydrophobic drugs.

**Figure 8:**
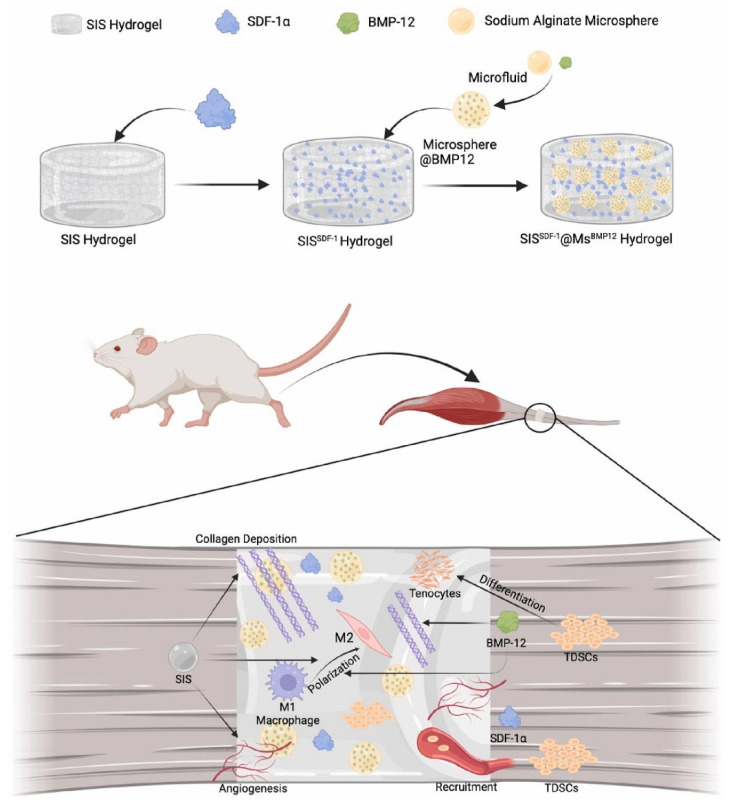
Schematic diagram of the preparation and function of SISSDF−1@MsBMP12 composite. Copyright Permission from Xie et al. [155] Biomaterials 2025.

Furthermore, compositing SA hydrogels with nanoparticles or other polymers enables the development of multi-level drug delivery systems for staged release of multiple drugs, which is significant for combination therapies in complex diseases. Xie et al. [155] developed a biphasic sustained-release composite material (SISSDF−1@MsBMP12) based on small intestinal submucosa hydrogel (SIS) and SA microspheres, which sequentially released stromal cell-derived factor-1α (SDF-1α) and bone morphogenetic protein-12 (BMP-12) to effectively recruit tendon-derived stem cells and promote their tenogenic differentiation. This material demonstrated significant immunomodulatory functions, ordered collagen deposition, and mechanical property recovery in in vivo and in vitro experiments, offering an innovative strategy to address challenges in tendon regeneration, such as insufficient cell recruitment and uncontrolled differentiation. Cardiac fibrosis secondary to myocardial infarction (MI) poses a significant challenge in heart disease management, necessitating innovative therapeutic strategies. Wu et al. [156] prepared an SA hydrogel (EVs-PAP@SA) loaded with bone marrow mesenchymal stem cell-derived extracellular vesicles (EVs) and p38α antagonist peptide (PAP) to intervene in fibrosis-inducing pathways in post-infarction myocardial tissue. The results demonstrated that this novel composite material effectively suppressed cardiac fibrosis formation, exhibiting strong anti-fibrotic effects (Figure 8). These multi-drug delivery systems exemplify how spatiotemporal control over the release sequence and rate of multiple therapeutic factors can synergistically address various aspects of disease pathogenesis, providing unprecedented capabilities for treating tissue regeneration disorders and complex chronic diseases.

### 4.2. Tissue Engineering and Regenerative Medicine

**Figure 9:**
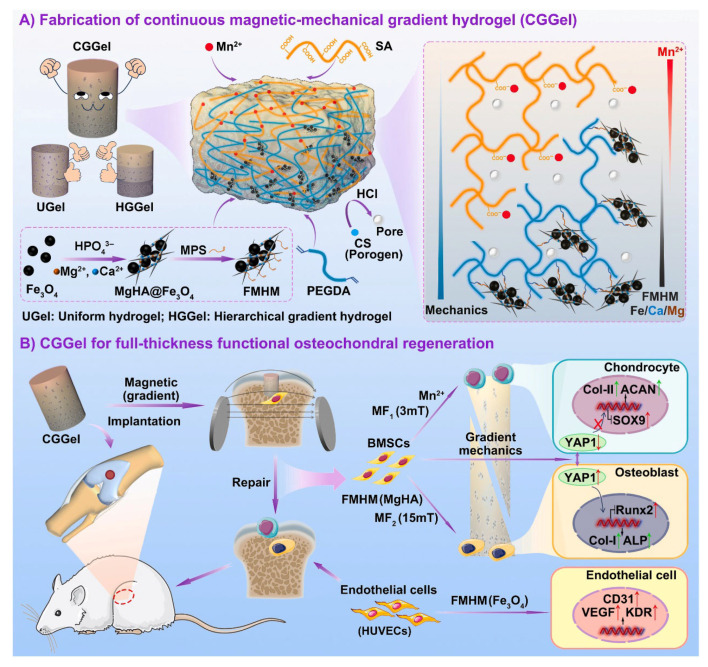
(**A**) Schematic diagram of the preparation of CGGel. (**B**) Schematic diagram of the mechanism by which CGGel promotes cartilage regeneration. Copyright Permission from Fan et al. [159] Bioactive Materials 2025.

SA-based hydrogels, with their three-dimensional network structure and cell-friendly properties, serve as ideal scaffold materials for tissue engineering and regenerative medicine [82]. In soft tissue repair, SA hydrogels can act as cell scaffolds, providing a favorable microenvironment for cell adhesion, proliferation, and differentiation. By compositing with HA [102], gelatin [105], or collagen [146,157], their mechanical properties and biocompatibility can be further enhanced. Significant progress has been made in the application of various SA hydrogels in cartilage repair. Kaviani et al. [158] developed an alginate-dendrimer hydrogel by covalently conjugating SA with fifth-generation polyamidoamine (PAMAM) dendrimers and incorporating 58S bioactive glass. The resulting novel hydrogel demonstrated excellent biocompatibility and mechanical properties for articular cartilage regeneration, with significantly improved tensile and compressive moduli, reduced degradation rates, and effective promotion of cell adhesion and sustained drug release, thus opening new avenues for tissue engineering. To address the issue of traditional layered gradient scaffolds, which are prone to delamination due to abrupt stress changes, rendering them ineffective for comprehensive osteochondral defect repair, Fan et al. [159] proposed a novel SA hydrogel with continuous magnetic-mechanical properties and gradients of multiple functional metal elements for functional osteochondral regeneration. They utilized a magnetic field to induce a gradient distribution of magnetic particles, producing the continuous mechanical gradient SA/PEGDA hydrogels (CGGEL) that mimic the natural mechanical gradient in osteochondral tissue, providing mechanical and magnetic gradient variations for full-layer osteochondral regeneration. Notably, following the secondary crosslinking of SA with Mn^2+^, a concentration gradient of Mn^2+^ was achieved, establishing an inverse gradient distribution of Mn^2+^, magnesium hydroxyapatite (MgHA), and Fe_3_O_4_ NPs. These components enhanced the chondrogenic and osteogenic differentiation of bone marrow mesenchymal stem cells (BMSCs) and promoted gene expression related to angiogenesis in human umbilical vein endothelial cells (HUVECs) (Figure 9).

By compositing SA hydrogels with HA or other inorganic components, the resulting composite materials not only exhibit excellent mechanical strength but also promote osteoblast adhesion and mineralization, thereby accelerating bone tissue regeneration [160,161]. Zhang et al. [162] developed an alginate hydrogel system containing Lycium barbarum polysaccharides to promote bone defect repair. This system enabled sustained release of active components in the bone defect area, effectively enhancing osteogenesis and angiogenesis, providing a potential avenue for bone tissue engineering and regenerative medicine. Cai et al. [146] investigated a novel titanium alloy scaffold coated with a deferoxamine (DFO)-functionalized alginate–collagen composite hydrogel, which enhanced the integration of metal implants with the bone interface by promoting osteogenesis and angiogenesis. Through in vitro cell experiments and in vivo animal model studies, the composite material was shown to significantly improve osseointegration, as evidenced by increased new bone formation, improved bone tissue maturity, and enhanced bone tissue penetration into the scaffold. Developing scaffolds with mechanical and electrical properties closely resembling those of cardiac tissue remains a challenge. Karimzadeh et al. [163] successfully prepared a GelMA/alginate/polypyrrole/graphene biocomposite hydrogel by incorporating polypyrrole and carboxylated graphene. This hydrogel-enhanced scaffold’s biocompatibility and biodegradability, while graphene increased its strength and electrical conductivity, offering a promising material with excellent biocompatibility and non-cytotoxicity for cardiac tissue engineering.

### 4.3. Biosensing and Diagnostics

**Figure 10:**
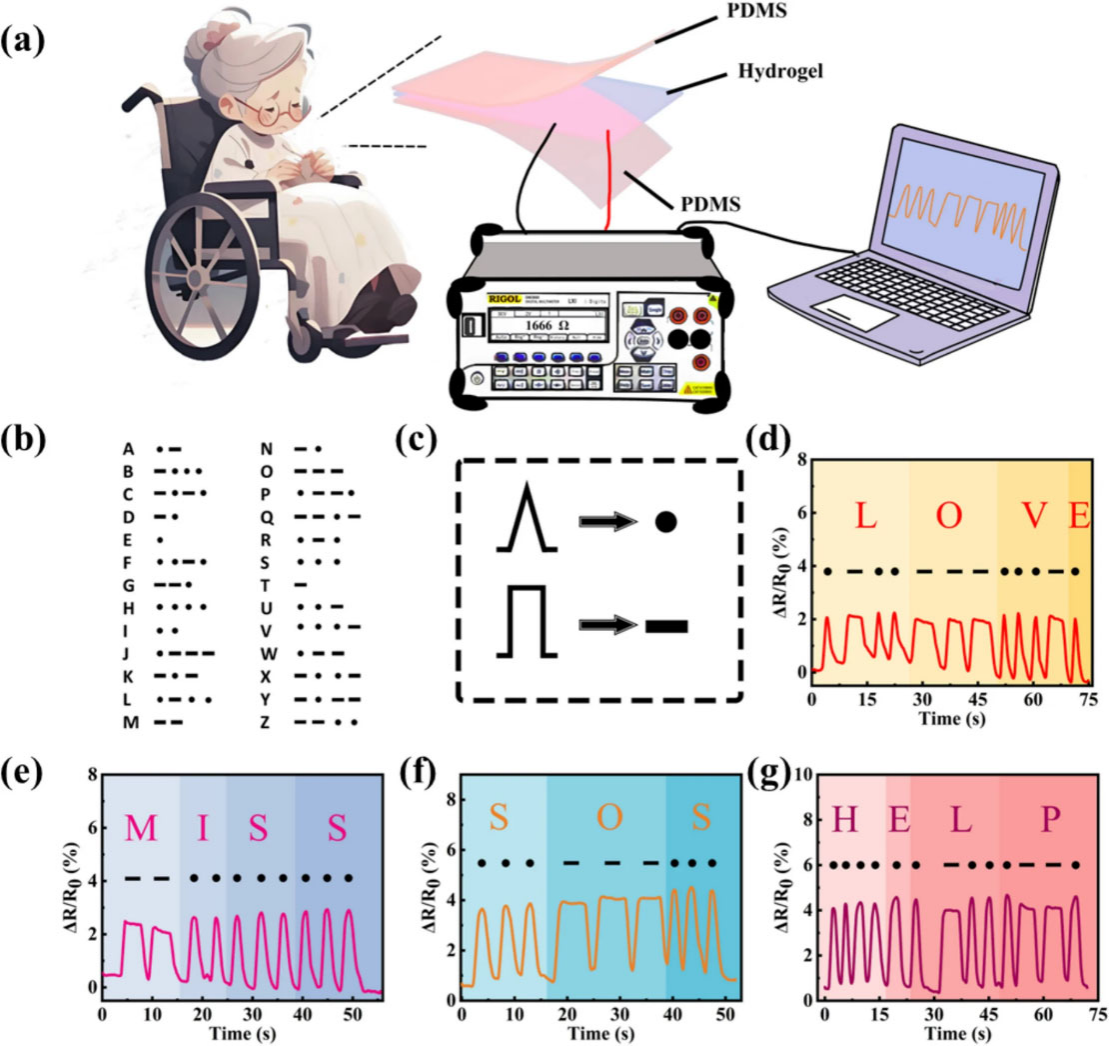
(**a**) Schematic diagram of PGS-Ca^2+^/LiCl hydrogel sensor’s precise recognition and high sensitivity. (**b**,**c**) Schematic diagram of the signal codes. (**d**–**g**) Morse code signals of different English words obtained through the PGS-Ca^2+^/LiCl hydrogel sensor. Copyright Permission from Wei et al. [148] Advanced Composites and Hybrid Materials 2025.

By compositing conductive materials such as graphene or carbon nanotubes, SA-based hydrogels can serve as functional materials for flexible biosensors [164,165]. Multifunctional hydrogels are widely applied in biosensing but face key challenges related to functional integration, environmental adaptability, and sustainability. To address the limitations of traditional hydrogels, such as insufficient mechanical strength, single functionality, non-recyclability, and poor dynamic response, Gu et al. [147] proposed a counterion-regulated differential metal ion coordination strategy, successfully developing a multifunctional hydrogel (SP-Mn+Xy−) with switchable modules, recyclability, and time-dependent encryption properties. By introducing metal ions (e.g., Fe^3+^ and Ca^2+^) and their corresponding counterions (e.g., Cl^−^ and Ac^−^), the regulatory effects of counterions on effective metal ion concentration, coordination kinetics, and network structure were systematically investigated. This hydrogel exhibited excellent mechanical properties (0.52 MPa tensile strength), a wide sensing range (300%), rapid response (114 ms), and anti-freezing capability (−20 °C). Through the reversible chelation of ethylenediaminetetraacetic acid (EDTA), efficient switching between conductive, adhesive, and sensing modules was achieved, with performance degradation below 17.95%. Dynamic encryption was achieved by utilizing the time-dependent coordination kinetics of Fe^3+^/Ca^2+^. The hydrogel also demonstrated high conductivity (15.1 S·m^−1^) and self-adhesion (9.5 kPa), making it suitable as a wearable sensor for motion tracking, voice recognition, and electrocardiogram monitoring. Wei et al. [148] developed a high-strength, highly stretchable, stress-sensing multifunctional ionic conductive hydrogel (PAM/Gelatin/SA-Ca^2+^/LiCl) by toughening PAM/gelatin semi-interpenetrating networks with SA. The PGS-Ca^2+^/LiCl hydrogel sensitively detected pressure changes, accurately monitoring human motion from vocal vibrations to complex deformations, showing significant potential for applications in electronic skin, wearable devices, and flexible electronics (Figure 10). Wang et al. [166] integrated hydroxypropyl methylcellulose (HPC) and MXene@PDA nanocomposite conductive materials into an SA hydrogel system through static self-assembly and calcium ion crosslinking strategies, successfully developing a conductive structural color hydrogel (CSCH) patch with dual photoelectrical signal sensing for precise diagnosis and effective treatment of myocardial infarction (MI). This hydrogel not only exhibited excellent electromechanical responsiveness and mechanochromic properties but also adhered closely to tissues. It provided real-time electrical signal feedback by altering relative resistance and displayed vivid visual data by adjusting the orientation of the liquid crystal phase, enabling digital and visual monitoring of subtle interfacial mechanical stimuli and spatiotemporal mechanical stimuli.

**Figure 11:**
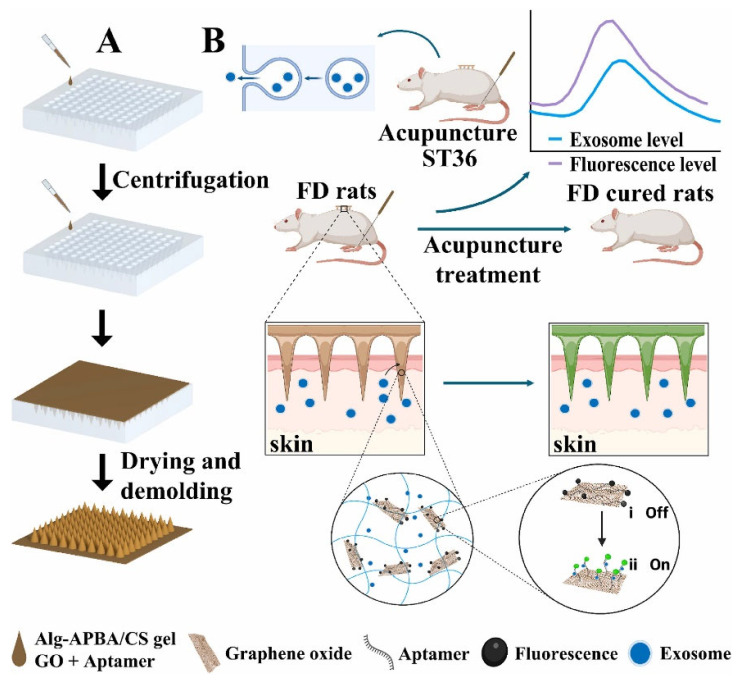
(**A**) Schematic diagram of the preparation of GOA-HMS. (**B**) Schematic diagram of exosome detection in interstitial fluid of the skin. Copyright Permission from Zhu et al. [165] Biosensors and Bioelectronics 2025.

SA-based hydrogels can serve as diagnostic platforms for capturing and detecting target molecules or cells. Gram-positive bacteria are the primary pathogens causing wound infections and suppuration. Liu et al. [167] developed a conductive bilayer hydrogel with excellent biocompatibility, constructed using dopamine-grafted hyaluronic acid (HA-DA), poly(N-isopropylacrylamide) (PNIPAM), and conductive poly(3,4-ethylenedioxythiophene)/poly(styrenesulfonate) (PEDOT: PSS). This hydrogel not only protects wounds during the healing process but also enables selective detection and treatment of Gram-positive bacterial infections. Additionally, it facilitates adaptive removal to prevent secondary damage, demonstrating significant potential for intelligent wound management. Wei et al. [168] incorporated Fe^2+^/Fe^3+^ redox pairs into nanofiber-reinforced SA hydrogels to develop a tailorable, breathable, and biocompatible “thermoelectric” dressing (TGC dressing). This dressing converts the temperature difference between the wound and the environment directly into electrical stimulation, significantly accelerating cell migration, angiogenesis, and collagen deposition. It also exhibits an antibacterial efficacy of >99.9%, low ion leakage, and wireless monitoring capabilities for temperature, exudate, and respiration, integrated with a wearable Bluetooth system, providing a clinically viable paradigm for next-generation zero-power intelligent wound treatment. Zhu et al. [165] developed a graphene oxide (GO)-aptamer hydrogel microneedle sensor (GOA-HMS) for real-time detection of exosomes in response to acupuncture treatment (point-of-care testing, POCT). The GOA-HMS was fabricated using micromolding technology and incorporated a composite hydrogel matrix of SA and GO as a nanofiller. The detection mechanism relies on a sensor formed by coupling graphene oxide with fluorophore-modified nucleic acid aptamers. The fluorescence intensity of GOA-HMS increased with the concentration of exosomes in interstitial fluid, demonstrating its potential as a sensitive detection platform (Figure 11). Wang et al. [169] have successfully developed a dual-responsive biopolymer nanocoating by integrating tannic acid-derived CDs with an alginate–zinc complex exhibiting aggregation-induced emission. This nanocoating demonstrates synergistically enhanced fluorescence and antibacterial properties, enabling both visual detection and active elimination of environmental and biological pollutants. It holds promising potential for broad applications in health monitoring, antimicrobial protection, and smart textiles.

## 5. Conclusions

SA-based hydrogels, as a widely available and high-performance natural polymer material, exhibit broad application prospects in the biomedical field due to their excellent biocompatibility, biodegradability, and versatile functionalization capabilities. By continuously expanding the application forms and functionalities of SA hydrogels, their multifunctionality in biomedical applications has been comprehensively demonstrated, spanning drug delivery, tissue engineering, wound repair, and biosensing. The multifunctionality and adaptability of SA hydrogels position them as key candidate materials for addressing complex medical challenges. These applications not only highlight the diversity of their material design but also provide efficient and safe solutions for tackling intricate medical problems.

In recent years, through performance optimization and composite modification, the mechanical properties, degradation rates, and functionalization levels of SA hydrogels have been significantly improved. However, challenges remain in areas such as insufficient mechanical strength, precise control of degradation behavior, stability in complex environments, and industrial-scale production. To overcome these challenges, future research should focus on intelligent material design, refinement of fabrication processes, and diversification of application scenarios. Additionally, through interdisciplinary collaboration and further advancement of clinical trials, the translation of SA hydrogels from laboratory research to clinical applications can be accelerated. Looking forward, several emerging strategies are poised to further advance the development and application of sodium alginate (SA)-based hydrogels. High-throughput screening approaches enable rapid evaluation of large libraries of SA formulations and crosslinking conditions, facilitating the identification of optimal compositions for specific biomedical applications. Meanwhile, machine learning-based material design provides a powerful tool to predict hydrogel properties, optimize formulation parameters, and accelerate the discovery of novel SA-based composites with tailored mechanical, biological, and drug release characteristics. In addition, scale-up manufacturing strategies, including automated microfluidic platforms and advanced 3D printing techniques, promise reproducible, high-throughput production of hydrogel constructs with precise architecture and batch-to-batch consistency. Integrating these approaches could significantly enhance the translational potential of SA hydrogels, enabling more efficient, predictable, and clinically relevant biomedical applications.

In summary, the research and application of SA hydrogels not only provide new insights for innovation in the field of biomedical materials but also offer efficient, cost-effective, and sustainable solutions for addressing current complex medical challenges. With continuous technological advancements and deeper exploration of applications, these materials are poised to play an increasingly significant role in the future of biomedical fields.

## Figures and Tables

**Table 1:** Comparison of physical, ionic and covalent crosslinking.

Property	Physical Crosslinking	Ionic Crosslinking	Covalent Crosslinking
Mechanical Strength	Low	Moderate	High
	Weak physical forces and entanglements are easily disrupted.	Strength depends on ion type and concentration.	High energy covalent bonds form a strong, stable 3D network with high load-bearing capacity.
Stiffness	Low	Highly Tunable	High (and Tunable)
	Soft and brittle, with low modulus.	Precisely adjustable via ion type, concentration, and crosslinking time.	Typically exhibits high elastic, adjustable via crosslinker density.
Toughness	Low	Moderate	High (but can be brittle)
	Prone to irreversible fracture with low energy absorption.	Exhibits energy dissipation via the reversible rupture and reformation of ionic bonds.	The network itself is often brittle.
Self-Healing	Excellent	Good	Poor (Typically Irreversible)
	Physical interactions can reversibly reassemble, allowing for rapid self-healing.	The dynamic nature of ionic bonds allows for inherent self-healing capability after damage.	Covalent bonds, once broken, are permanently lost, no inherent self-healing ability. (Excluding dynamic reversible covalent crosslinking.)
Stability	Poor (Sensitive)	Moderate (Ion-sensitive)	Excellent (Stable)
	Highly sensitive to temperature, pH, and ionic strength; very unstable.	Susceptible to ion exchange and dissolution in high ionic strength solutions or chelating agents.	High chemical stability, resistant to solvents, ionic erosion, and degradation.
Typical Applications	Drug delivery	Cell encapsulation, tissue engineering scaffolds, wound dressings	Applications requiring long-term mechanical stability, e.g., cartilage repair

**Table 2:** Performance optimization strategies for SA-based hydrogels.

Main Constituents	Gelation Mechanism	Preparation Form	Optimization Strategy	Optimized Performance	Applications	Ref.
CS, SA, NaCl	Ionic crosslinking	Scaffold	Combining polymers	High hydrophilicity	Tissue engineering and regenerative medicine	[32]
OSA, carboxymethyl chitosan (CMCS)	Covalent crosslinking	Scaffold	Combining polymers	Self-healing	Drug delivery	[12]
SA, CaCl_2_, crystalline nanocellulose (CNC)	Ionic crosslinking	Microsphere	Combining polymers	Increased swelling degree	Drug delivery	[95]
cellulose nanofibers (CNF), SA, CaCl_2_	Ionic crosslinking	Microsphere	Introducing nanomaterials	Green, low-toxicity, biodegradable, and good mechanical properties	Drug delivery	[26]
CNC, SA, Ca^2+^	Ionic crosslinking	General hydrogel	Introducing nanomaterials	Increased density of the gel network	Drug delivery	[96]
OSA, acrylamide-modified CS, polyacrylamide (PAM)	Covalent and physical crosslinking	General hydrogel	Constructing dynamic cross-linking networks	High toughness and self-healing properties	Biosensing and diagnostics	[97]
Ag, Fe_3_O_4_, SA, CaCl_2_	Ionic crosslinking	Microsphere	Intelligent responsive modification	Antibacterial and magnetic-/pH-sensitive	Drug delivery	[98]
polyethylene glycol (PEG), polypropylene glycol, polycaprolactone, SA, Ca^2+^	Ionic and physical crosslinking	General hydrogel	Intelligent responsive modification	Temperature and Ca^2+^ response	Tissue engineering and regenerative medicine	[99]
SA, CaCO_3_, QK and RGD peptide	Ionic crosslinking	General hydrogel	Surface functionalization	Enhance adhesion and multiple aspects of MSC regenerative potential	Tissue engineering and regenerative medicine	[100]
SA, cystamine, pluronic F127,	Physical crosslinking	General hydrogel	Surface functionalization	Excellent cytocompatibility and effective antimicrobial activity	Tissue engineering and regenerative medicine	[101]
SA, HA, bioactive glass (BG), poly (lactic-co-glycolic acid), kartogenin	Covalent crosslinking	General hydrogel	Drug loading and controlled release design	improving tissue infiltration and sequentially delivering bioactive substances	Tissue engineering and regenerative medicine	[102]
Zn^2+^, SA, hyperbranched poly(β-amino esters), TNF-α siRNA	Ionic and physical crosslinking	General hydrogel	Drug loading and controlled release design	Inducing reprogramming of immunometabolism	Drug delivery	[103]
SA, BG, deferoxamine (DFO)	Covalent crosslinking	General hydrogel	Regulating degradability	Faster degradation and better tissue infiltration	Tissue engineering and regenerative medicine	[104]

**Table 3:** Correspondence between optimization strategies and applications of SA-based hydrogels.

Applications	Key Requirements	Optimization Strategies	Example Ref.
Drug Delivery	Controlled release, targeting, biocompatibility	Intelligent responsive modification, Drug loading design, Nanomaterial reinforcement	[98,123,140]
Tissue Engineering	Mechanical strength, cell adhesion, biodegradability	Combining polymers, Surface functionalization, Dynamic cross-linking	[32,100,127]
Regenerative Medicine	Bioactivity, vascularization, tissue integration	Surface functionalization, Drug loading, Degradation regulation	[102,104,146]
Biosensing and Diagnostics	Conductivity, responsiveness, stability	Intelligent responsive modification, Nanomaterial reinforcement	[131,147,148]

## Data Availability

No new data were created or analyzed in this study. Data sharing is not applicable to this article.
